# Screening a newly developed common bean germplasm with improved resistance to ashy stem blight in multiple environments

**DOI:** 10.3389/fpls.2022.1052398

**Published:** 2022-11-23

**Authors:** Diego M. Viteri, Angela M. Linares-Ramírez

**Affiliations:** ^1^ Department of Agro-environmental Sciences, University of Puerto Rico, Isabela Research Substation, Isabela, Puerto Rico; ^2^ Department of Agro-environmental Sciences, University of Puerto Rico, Lajas Research Substation, Lajas, Puerto Rico

**Keywords:** Common bean (*Phaseolus vulgaris* L.), *Macrophomina phaseolina* (Tassi) Goid., germplasm, partial resistance, fungal pathogen

## Abstract

Ashy stem blight (ASB) caused by the necrotrophic fungus *Macrophomina phaseolina* (Tassi) Goidanich is an important disease in common bean (*Phaseolus vulgaris* L.) in the Americas and worldwide. Low to intermediate levels of ASB resistance exist in cultivated and landrace genotypes of the common bean and the tertiary gene pool. However, cultivars with higher levels of resistance are not yet available. Our objectives were to 1) pyramid higher levels of resistance from multiple parent populations within the primary gene pool and 2) compare the response of the newly developed breeding lines (BL) with known sources of resistance. The BL UPR-Mp-22, UPR-Mp-34, UPR-Mp-42, and UPR-Mp-48, known sources of resistance, and susceptible checks were inoculated twice per plant with the PRI21 *M. phaseolina* isolate in the greenhouse and field trials conducted in Isabela and Lajas, Puerto Rico. None of the genotypes tested were resistant (mean scores 1–3). However, the new black UPR-Mp-42 and white UPR-Mp-48 BL had an intermediate response (mean scores 4–6) compared to white common bean genotypes ‘Bella,’ NY6020-4, and ‘Verano’ and black bean TARS-MST1 that were susceptible (scores ≥7) in all environments. Andean genotypes A 195, PRA154, PRA155, and UPR-Mp-22 were intermediate in the greenhouse. In contrast, UPR-Mp-34 had significantly lower scores than BAT 477 that had a susceptible reaction in the greenhouse in Isabela and in the field in Lajas and SEA 5 that was susceptible in all environments. These new BL possess an enhanced ASB resistance and may be used to improve common bean cultivars or germplasms of different market classes.

## Introduction

Ashy stem blight (ASB) caused by the fungus *Macrophomina phaseolina* (Tassi) Goidanich is an important disease in the common bean (*Phaseolus vulgaris* L.) in tropical and subtropical regions in the Americas and elsewhere (Singh and Schwartz, 2010; Ambachew et al., 2021). This plant pathogen affects roots and all aerial plant parts causing damping off, root rots, stem blight, leaf burning, and premature senescence symptoms (Islam et al., 2012; Kaur et al., 2012; Marquez et al., 2021). Yield losses over 60% in susceptible common bean genotypes were reported (Mayek et al., 2003; Kaur et al., 2012; Viteri and Linares, 2022). The fungus is endemic and seed-transmitted, and its sclerotia can survive in the soil up to 15 years (Marquez et al., 2021). Furthermore, different levels of aggressiveness between *M. phaseolina* isolates were reported (Miklas et al., 1998a; Mayek et al., 2001a; Viteri and Linares, 2017). Disease avoidance mechanisms such as genotypes with upright growth habit may prevent severe ASB infections in the field (Viteri and Linares, 2022). However, the use of germplasm with higher levels of resistance is crucial, since fungicide applications do not provide an adequate control for this pathogen (Singh and Schwartz, 2010; Marquez et al., 2021).

Low to higher levels of ASB resistance have been reported in the common bean and tepary bean (*Phaseolus acutifolius* A. Gray) (Miklas et al., 1998a; Mayek et al., 2001b; Pastor-Corrales and Abawi, 1988; Viteri and Linares, 2017). Furthermore, the resistance to ASB can be inherited qualitatively or quantitatively depending of the genotypes used, plant organ evaluated, environment, virulence of the isolate, and/or methodology of the screening used (Olaya et al., 1996; Miklas et al., 1998b; Viteri and Linares, 2019). For instance, common bean genotypes A 195, ‘Badillo,’ NY6020-4, PRA154, and PRA155 were reported with partial ASB resistance (Viteri and Linares, 2017). Likewise, BAT breeding lines (BL) (e.g., BAT 85, 332, 477, 1651), black common bean genotypes, ‘Negro Tacaná,’ ‘Negro Perla,’ TARSMST-1, and XAN 176, red-mottled cultivars ‘PC 50’ and ‘San Cristobal 83,’ and landraces from the Dominican Republic were reported to have intermediate and higher levels of resistance (Echavez-Badel and Beaver, 1987; Pastor-Corrales and Abawi, 1988; Viteri and Linares, 2017). Also, tepary bean accessions Mex-114, PI 440806, and PI 321637 were reported with higher levels of ASB resistance (Miklas et al., 1998a). Within the common bean gene pool, five quantitative trait loci (QTL) located on chromosomes Pv04, Pv06, Pv07, and Pv08 and derived from XAN 176 conferred field resistance in the Dorado/XAN 176 common bean population (Miklas et al., 1998b). Likewise, Mayek et al. (2009) and Méndez et al. (2017) identified that two complementary genes and nine QTL on Pv03, Pv05, Pv06, Pv08, Pv09, and Pv10 chromosomes derived from BAT 477 provided field resistance in the UI-114/BAT 477 recombinant inbred line population. Conversely, two complementary genes, namely, *Mp-1* and *Mp-2*, conferred resistance in the A-70/BAT 477 cross in a controlled environment, and these genes were also derived from BAT 477 (Olaya et al., 1996). More recently, Viteri and Linares (2019) reported that two complementary recessive genes and one recessive gene conferred resistance to ASB in the PC 50/’Othello’ and Badillo/PR1144-5 populations, respectively, in the greenhouse by the cut-stem method. These genes were apparently derived from Andean genotypes Badillo and PC 50. In the same study, one dominant gene conferred resistance in the A 195/PC 50 population.

Breeding for ASB resistance has been limited compared to other necrotrophic fungi such as *Sclerotinia sclerotirum* L. de Bary (the causal agent of white mold) from which diverse germplasms have been released in the last years (Schwartz and Singh, 2013; Singh et al., 2016; Singh et al., 2017). The cream-colored BAT 477 BL and Negro Tacaná and red-mottled San Cristobal 83 cultivars were the only genotypes used to develop the common bean germplasms with resistance to soilborne fungi, including *M. phaseolina*, and drought and heat tolerance. For example, SEA 3 and SEA 5 BL were developed from the multiple parent crosses BAT 477/San Cristobal 83//BAT 93/‘Jalo EEP 558’ and BAT 477/San Cristobal 83//‘Guanajuato 31’/‘Rio Tibagi,’ respectively (Singh et al., 2001). Likewise, Porch et al. (2012) developed the TARS-MST1 black common bean with ASB resistance from the single cross Negro Tacaná/VAX 6. However, the development of a new germplasm combining genotypes from the Andean (e.g., A 195, PC 50, PRA154, and PRA155) and Middle American races of the common bean is necessary to increase the levels of ASB resistance. The objectives of this research were to 1) develop BL with improved resistance to ASB and 2) compare the response of the newly developed BL with known sources of resistance.

## Materials and methods

### Development of breeding lines with pyramided ashy stem blight resistance

The UPR-Mp-22 BL was generated from A 195/PC 50//PRA155, while UPR-Mp-34 was selected from A 195/PC 50//SEA 5 common bean crosses. The UPR-Mp-42 and UPR-Mp-48 BL were developed from the cross BAT 477/NY6020-4//PRA154. Of the seven parents used in these triple crosses, Andean common beans A 195 (Singh et al., 2007), PC 50 (Saladin et al., 2000), NY6020-4 (Miklas and Delorme, 2003), PRA154, and PRA155 (Singh et al., 2017) possess growth habit type I (Singh, 1982) and have low to partial levels of ASB resistance (Viteri and Linares, 2017; Viteri et al., 2019). Likewise, these Andean genotypes were reported to have intermediate to higher levels of resistance to white mold (Viteri et al., 2015; Singh et al., 2017). BAT 477 (Viteri and Linares, 2022) and SEA 5 (Singh et al., 2001) have growth habit type III (Singh, 1982) and were reported with low to high levels of resistance to *M. phaseolina* (Pastor-Corrales and Abawi, 1988; Olaya et al., 1996; Singh et al., 2001; Méndez et al., 2017; Viteri and Linares, 2017; Viteri et al., 2019).

### Ashy stem blight screening in the greenhouse

An average of 55 F_1_ seeds of A 195/PC 50 and BAT 477/NY6020-4 were developed at the Isabela Research Substation at the University of Puerto Rico in January 2017. Four cycles of ASB screening from the F_1_ to F_4_ for both populations were conducted in May and September 2017 and February and September 2018 in greenhouses at the Isabela and Lajas Research Substations. A completely random design without replication was used in each cycle. One seed of each genotype was planted in 16-cm-diameter plastic pots containing Pro-Mix BX at pH 5.9. Plants were inoculated at the fourth internode by the cut-stem method (Viteri and Linares, 2017; Viteri et al., 2019) with the PRI16 isolate for the screening of the F_1_ and F_2_ and with the PRI18 isolate for the F_3_ and F_4_. Only resistant plants (scores ≤3; Viteri and Linares, 2017; see below) to both *M. phaseolina* isolates were selected at harvest and advanced to the next generation by the pedigree method. The F_4:5_ resistant plants of the A 195/PC 50 population were crossed with SEA 5 and PRA155 genotypes, while the BAT 477/NY6020-4 F_4:5_ resistant plants were crossed with the Andean common bean PRA154 in February 2019 in Isabela. Resistant plants of PRA154, PRA155, and SEA 5 BL to one inoculation of PRI18 isolate were also selected to develop the triple crosses by the gamete selection breeding method (Singh, 1994). An average of 70 F_1_ seeds were produced for each population. One seed of the F_1_ for the crosses A 195/PC 50//PRA155, A 195/PC 50//SEA 5, and BAT 477/NY6020-4//PRA154 was planted in a plastic pot as mentioned previously, and each plant was inoculated with the PRI19 isolate in May 2019 in Isabela, while each plant from the F_2_ to F_5_ was inoculated twice with the PRI19 and PRL19 isolates (similar as the methodology described in the comparative greenhouse and field trials section) in September 2019 and January, May, and September 2020 in Lajas. Resistant plants from each cross were selected and advanced in each generation by the pedigree method in the greenhouse. The F_5:6_ resistant plants of each population were used to develop the UPR-Mp-22, UPR-Mp-34, UPR-Mp-42, and UPR-Mp-48 BL. Furthermore, selection of plants with cream mottled (‘cranberry’ type), cream, black, and white seed coat colors was conducted for UPR-Mp-22, UPR-Mp-34, UPR-Mp-42, and UPR-Mp-48 BL, respectively, from the F_3_ to F_6_. It is important to mention that all greenhouses had the optimum environment (i.e., high mean day temperatures >26°C and moisture ranged from 50% to 70% after the inoculation; Pastor-Corrales and Abawi, 1988; Mayek et al., 2002; Viteri and Linares, 2022) to promote a higher ASB pressure. In addition, susceptible checks such as white bean ‘Beníquez’ and pinto ‘Othello’ were used in all of the greenhouse screening. In fact, these cultivars were highly susceptible (mean scores between 8 and 9) to all of the *M. phaseolina* isolates used.

### Comparative greenhouse and field trials

The newly developed UPR-Mp-22, UPR-Mp-34, UPR-Mp-42, and UPR-Mp-48 BL with partial resistance to ASB along with their resistance donor parents, black BL TARS-MST1 and XAN 176 reported with resistance to *M. phaseolina* (Miklas et al., 1998b; Porch et al., 2012), and susceptible white cultivars ‘Bella’ and ‘Verano’ (Viteri and Linares, 2017; Viteri and Linares, 2019) were evaluated in the greenhouse and the field in Isabela and Lajas in 2021. One inoculation of the PRI21 *M. phaseolina* isolate at the fourth internode was conducted at ~21 days after planting. A second inoculation was carried out in a lateral branch close to the base of the main stem 10 days later. A 200-μl Eppendorf tip stacked with four mycelial plugs from 48-h-old *M. phaseolina* culture grown at 28°C on potato dextrose agar at 12:12 h (light: dark) was used for each inoculation. A randomized complete block design with four and three replications were used for the greenhouse and field trials, respectively, conducted in April and May in Isabela and in February and September 2021 in Lajas. In the greenhouse screening, four plants of each genotype (i.e., two seeds planted in a 16-cm-diameter plastic pot containing Pro-Mix BX) per replication were used under the environmental conditions described in the previous section. For the field trials, 20 seeds of each genotype were planted in rows of 1.5 m per replication. The distance between rows (genotypes) were 0.8 m. Isabela is located at 126 m above sea level and has Oxisol soils, mean annual precipitation of 1,592 mm, and an average relative humidity of 70% (Muñoz et al., 2018). Lajas is located at 9 m above sea level and has soils belonging to the Mollisol and Vertisol orders, an annual precipitation of 1,143 mm, and an average relative humidity of 80% (Muñoz et al., 2018). Both locations had prolonged periods of high temperatures (>27°C) (Viteri and Linares, 2022) at the time of inoculations that promoted a severe ASB pressure in the field. Ten plants on the central rows were inoculated twice with the PRI21 isolate following the methodology described previously.

**Figure 1:**
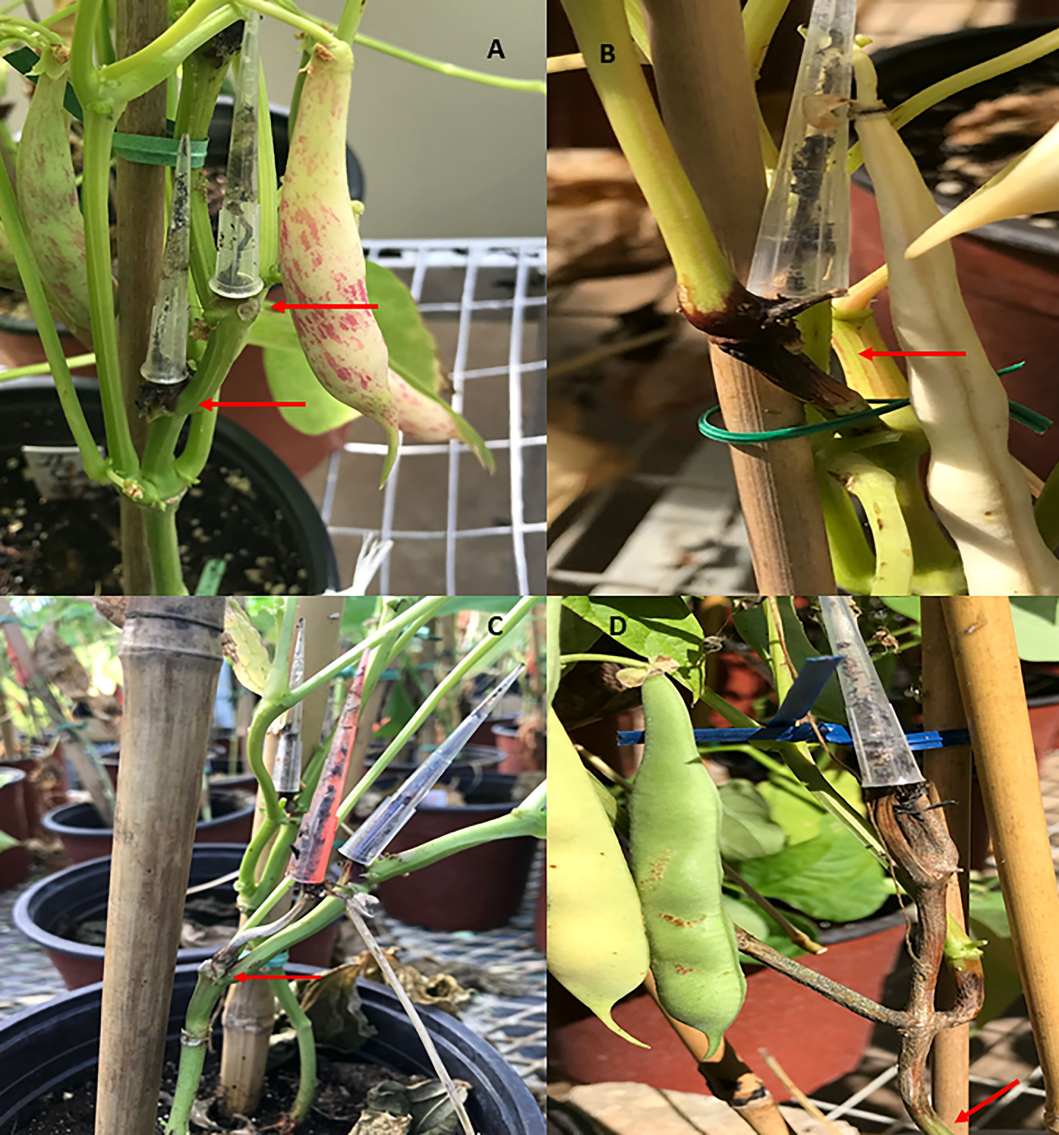
The ashy stem blight disease was scored at 42 and 21 days post inoculation in the greenhouse and the field, respectively, using a 1–9 scale, where 1–3 = resistant, 4–6 = intermediate, and 7–9 = susceptible. **A**, score 3; **B**, score 5; **C**, score 6; and **D**, score 9.

The disease severity was evaluated at 42 and 21 days after the second inoculation in the greenhouse and the field, respectively. A 1–9 scale was used, where 1 = no sign of *M. phaseolina* infection, 3 = the fungus did not pass the first node above or below the point of the inoculation, 5 = the pathogen passed the first node above or below the point of the inoculation and infected the internode no more than the 50%, 6 = *M. phaseolina* reached the second node above or below the point of the inoculation, 7 = the fungus infection reached the second node above or below the stem, and 9 = the pathogen passed the third node above or below the point of the inoculation causing plant death in most of the cases (Viteri and Linares, 2017). The higher scores between the two inoculations conducted in each plant were used to measure the response of the genotypes to PRI21 isolate and for the statistical analysis. Furthermore, genotypes with mean scores of 1–3 were considered resistant, 4–6 were intermediate, and 7–9 were susceptible (**Figure 1**; Viteri and Linares, 2017).

### Statistical analysis

A combined analysis of variance was performed for the ASB severity to determine the effect of the genotype, location, and their interactions for the greenhouse and the field. A mixed model was used for data analysis where the common bean genotypes and locations were treated as fixed effects, while replications were considered as random effects (McIntosh, 1983). Also, Bartlett’s test for homogeneity of variances (Cordeiro, 1983) was performed for the severity in each location and environment, and a Fisher’s least significant difference at *P* ≤ 0.05 was calculated to discriminate differences among genotypes. Data were analyzed using SAS 9.4 PROC GLM (SAS Institute, 2012). Furthermore, the disease range and percentages of resistant plants for each genotype were also calculated in the greenhouse and the field.

## Results

Mean squares for the genotype and the interaction genotype × location were significant (*P* ≤ 0.05) for the ASB severity in the greenhouse and the field. Conversely, there were no significant differences (*P* ≥ 0.05) for location for the two environments (**Table 1**). Variances for the data analyzed from the two locations were not homogeneous in the greenhouse (*χ*² = 16.16; *P* ≤ 0.0001) and the field (*χ*² = 15.31; *P* ≤ 0.0001), and therefore, the data for the ASB severity were presented separately in each location.

**Table 1:** Combined analysis of variance for ashy stem blight [caused by *Macrophomina phaseolina* (Tassi) Goidanich] severity for 15 common bean (*Phaseolus vulgaris* L.) genotypes evaluated in the greenhouse and the field in Isabela and Lajas, Puerto Rico, in 2021.

Source	df	df	Mean squares	
	Greenhouse	Field	Severity (Greenhouse)	Severity (Field)
Location (L)	1	1	7.01^ns^	3.03^ns^
Replication (Location)	6	4	9.78*	7.49***
Genotype (G)	14	14	69.45***	25.25***
G × L	14	14	7.37*	7.01***
Error	444	866	4.29	3.52

*Significant at the 0.05 probability level. *** Significant at the 0.01 probability level. ns, nonsignificant.

Resistant genotypes (scores ≤3) were not identified in this study. In fact, the disease range in each genotype varied from resistant to susceptible plants (**Table 2**). Nonetheless, the UPR-Mp-22, UPR-Mp-34, PRA154, and PRA155 genotypes had >35% of resistant plants to PRI21 *M. phaseolina* isolate in the greenhouse in Isabela and Lajas (**Table 2**). In contrast, UPR-Mp-22 reached close to 30% of resistant plants in the field in both locations, while UPR-Mp-42 and PRA155 had >55% of resistant plants in the field evaluation in Lajas (**Table 2**).

**Table 2:** Disease range and percentages of ashy stem blight-resistant plants for four pyramided common bean (*Phaseolus vulgaris* L.) breeding lines, their parents/known sources of resistance, and susceptible checks evaluated against PRI21 *Macrophomina phaseolina* (Tassi) Goidanich isolate at 42 and 21 days after inoculation in the greenhouse and the field in Isabela and Lajas, Puerto Rico, in 2021.

Genotype	Greenhouse					Field					
	Isabela (April)		Lajas (February)			Isabela (May)			Lajas (September)		
	Disease range	Resistant plants (%)	Disease range	Resistant plants (%)	Mean (%)	Disease range		Resistant plants (%)	Disease range	Resistant plants (%)	Mean (%)
*New breeding lines with partial resistance to ashy stem blight*											
UPR-Mp-22	2-9	43.8	3-6	62.5	53.2	3-9		33.3	2-9	30.0	31.7
UPR-Mp-34	2-9	68.8	3-9	56.3	62.6	3-9		10.0	2-9	63.3	36.7
UPR-Mp-42	2-9	43.8	3-9	37.5	40.7	3-9		30.0	3-9	16.7	23.4
UPR-Mp-48	2-9	43.8	3-9	56.3	50.1	3-9		16.7	2-9	20.0	18.4
*Parental genotypes and breeding lines reported with resistance to ashy stem blight*											
A 195	2-9	56.3	2-9	37.5	46.9	2-9		26.7	3-9	10.0	18.4	
BAT 477	3-9	6.3	4-9	0.0	3.2	3-9		6.7	4-9	0.0	3.4	
NY6020-4	3-9	18.8	3-9	6.3	12.6	3-9		3.3	3-9	3.3	3.3	
‘PC 50’	2-9	43.8	2-9	25.0	34.4	3-9		16.7	3-9	10.0	13.4	
PRA154	2-9	68.8	3-7	37.5	53.2	3-9		23.3	2-9	40.0	31.7	
PRA155	3-9	37.5	3-7	56.3	46.9	3-9		16.7	2-7	60.0	38.4	
SEA 5	3-9	12.5	3-9	18.8	15.7	4-9		0.0	4-9	0.0	0.0	
TARS-MST1	3-9	25.0	7-9	0.0	12.5	4-9		0.0	4-9	0.0	0.0	
XAN 176	3-9	6.3	4-9	0.0	3.2	3-9		16.7	3-9	6.7	11.7	
*Susceptible checks*											
‘Bella’	3-9	18.8	4-9	0.0	9.4	4-9	0.0		6-9	0.0	0.0
‘Verano’	3-9	6.3	7-9	0.0	3.2	4-9	0.0		5-9	0.0	0.0
Mean	…	33.4	…	26.3	29.9	…	13.3		…	17.3	15.3

With regard to the ASB severity, UPR-Mp-42 and UPR-Mp-48 had an intermediate response (4–6) compared to white common bean genotypes Bella, NY6020-4, and Verano and black bean TARS-MST1 that were susceptible (scores ≥7) in all environments. XAN 176 had an intermediate response in the evaluations conducted in Isabela; however, it was susceptible in Lajas (**Table 3**). The overall mean scores of the BL UPR-Mp-22 and Andean genotypes A 195, PRA154, and PRA155 were intermediate in all environments. In contrast, UPR-Mp-34 had significantly lower scores than BAT 477 that had a susceptible reaction in the greenhouse in Isabela and in the field in Lajas and SEA 5 that was susceptible in all environments (**Table 3**).

**Table 3:** Growth habit, seed color, and ashy stem blight mean scores for four pyramided common bean (*Phaseolus vulgaris* L.) breeding lines, their parents/known sources of resistance, and susceptible checks evaluated against PRI21 *Macrophomina phaseolina* (Tassi) Goidanich isolate at 42 and 21 days after inoculation in the greenhouse and the field in Isabela and Lajas, Puerto Rico, in 2021.

Genotype	Growth habit^a^	Seed color	Greenhouse			Field			
			Isabela April	Lajas February		Isabela May	Lajas September		
			Severity		Mean	Severity		Mean	
*New breeding lines with partial resistance to ashy stem blight*									
UPR-Mp-22	I	Cream-mottled	4.6	3.8	4.2	5.0	4.5	4.8	
UPR-Mp-34	III	Cream-colored	3.9	3.9	3.9	6.0	4.1	5.0	
UPR-Mp-42	I	Black	5.3	5.4	5.3	5.0	4.9	5.0	
UPR-Mp-48	I	White	4.2	4.4	4.3	5.9	4.7	5.3	
*Parental genotypes and breeding lines reported with resistance to ashy stem blight*									
A 195	I	Beige opaque	3.8	4.6	4.2	5.7	6.4	6.1	
BAT 477	III	Cream-colored	7.1	6.4	6.8	6.4	7.3	6.8	
NY6020-4	I	White	7.0	7.3	7.1	6.7	7.8	7.3	
‘PC 50’	I	Red-mottled	4.1	4.8	4.5	6.3	6.7	6.5	
PRA154	I	Beige-mottled	4.1	4.4	4.3	5.1	4.6	4.8	
PRA155	I	Cream with black spots	5.9	4.0	5.0	6.4	3.9	5.2	
SEA 5	III	Cream-colored	7.0	6.6	6.8	7.1	7.7	7.4	
TARS-MST1	II	Black	7.0	8.5	7.8	7.3	8.7	8.0	
XAN 176	III	Black	6.2	7.1	6.6	6.1	7.5	6.8	
*Susceptible checks*								
‘Bella’	II	White	7.1	7.6	7.4	7.0	8.6	7.8	
‘Verano’	II	White	7.0	9.0	8.0	7.5	8.0	7.8	
Mean	…	…	5.6	5.9	5.8	6.2	6.4	6.3	
LSD (*P* ≤ 0.05)	…	…	1.5	1.2	1.0	1.0	0.9	0.8	

a Growth habit, where I = determinate upright, II = indeterminate upright, III = indeterminate prostrate semiclimbing (Singh, 1982).

Ashy stem blight disease severity was scored on a 1–9 scale, where 1–3 = resistant, 4–6 = intermediate, and 7–9 = susceptible (Viteri & Linares, 2017).

## Discussion

Genetics and breeding for resistance to ASB disease have been conducted for more than 20 years in the United States and worldwide (Olaya et al., 1996; Miklas et al., 1998b; Mayek et al., 2001b; Méndez et al., 2017; Pastor-Corrales and Abawi, 1988; Viteri and Linares, 2019). However, common bean BL and cultivars with enhanced levels of resistance are not yet available. One of the reasons may be because the breeding against ASB has been focused only on Middle American beans that possess tolerance to heat and drought (Urrea et al., 2009; Beebe et al., 2013; Ambachew et al., 2021) and the severity of *M. phaseolina* increases in these environments (Frahm et al., 2004; Mayek et al., 2002). In fact, BAT 477 and Negro Tacaná were used as donor parents to improve the resistance to *M. phaseolina* and tolerance to the aforementioned abiotic stresses in the SEA 5 and TARS-MST1 BL, respectively (Singh et al., 2001; Porch et al., 2012). Unfortunately, these two genotypes were susceptible to ASB in this research and a previous study using mechanical inoculations (Viteri et al., 2019). Thus, QTL associated with tolerance to heat and drought may not necessarily provide or have a major effect against *M. phaseolina*. Further research is necessary to tag and identify new resistant QTL in Andean genotypes as the new QTL on Pv07 and Pv09 chromosomes derived from PRA154 and PC 50, respectively (Viteri, non-published information). Furthermore, the presence of avoidance mechanisms in some genotypes (e.g., TARS-MST1, Bella, and Verano) (Viteri and Linares, 2022) and the uneven dispersion of *M. phaseolina* inoculum in the field (Echavez-Badel and Beaver, 1987) may cause a lower ASB severity or an escape under natural infections and, thus, a misidentification of resistant genotypes.

Our emphasis in this study was to obtain an enhanced ASB resistance in BL developed from multiple parent crosses between the common bean races combining the pedigree and gamete selection techniques (Singh, 1994). In fact, our results and previous studies (Viteri and Linares, 2017; Viteri and Linares, 2019) showed that the Andean beans had higher levels of resistance than Middle American genotypes. Furthermore, the improved resistance in the new BL (mostly derived from the Andean race) was significant compared with their counterparts of the same market classes. For instance, the genetic gain for ASB resistance of the new UPR-Mp-34 BL (A 195/PC 50//SEA 5) varied from 27% to 33% compared to BAT 477 and SEA 5, respectively, in the field, while it was close to 42% in the greenhouse. Likewise, UPR-Mp-42 reached a genetic gain for resistance to *M. phaseolina* close to 23% compared with XAN 176 in both environments, while these values increased to 32% and 38% compared to TARS-MST1 in the greenhouse and the field, respectively. Within the white beans, the most important market class in Puerto Rico (Beaver et al., 2020), UPR-Mp-48 (BAT 477/NY6020-4//PRA154), had a genetic gain for resistance to this fungus between 42% and 50% in the greenhouse and 32% in the field than cultivars Bella and Verano. To the best of our knowledge, UPR-Mp-48 is the first white bean germplasm with intermediate resistance to ASB and determinate growth habit Type I (Singh, 1982). Both of these traits should be introgressed into Bella cultivar that possesses indeterminate growth habit Type II and resistance to the *Bean common mosaic virus*, *Bean common mosaic necrosis virus*, *Bean golden yellow mosaic virus*, common bacterial blight [caused by *Xanthomonas campestris* pv. *phaseoli* Smith (Dye)], and web blight [caused by the fungus *Thanatephorus cucumeris* (Frank). Donk (anamorph: *Rhizoctonia solani* Kühn)] (Beaver et al., 2018). With this, new cultivars for mechanical harvesting and with resistance to multiple diseases can be developed. Likewise, UPR-Mp-42 may be used to incorporate genes/QTL for ASB resistance and determinate growth habit Type I (Singh, 1982) into susceptible black bean genotypes (e.g., ‘Zorro’; Viteri and Linares, 2017) or TARS-MST1 that also possesses partial resistance to common bacterial blight (Kelly et al., 2009; Porch et al., 2012).

With regard to the crosses between Andean common beans, there was not a significant genetic gain for resistance of UPR-Mp-22 (A 195/PC 50//PRA155) compared to the reaction of its parents and PRA154 separately; in general, an intermediate response was observed. However, the developed UPR-Mp-22 belongs to the cranberry type, considered an important commercial market class in Canada and the United States (Miklas and Riley, 2012). Thus, this BL may be used to improve susceptible cultivars in this market class. Moreover, a new set of crosses between UPR-Mp-22 and the Andean beans Badillo, PRA154, and VA 19 reported with partial resistance to ASB (Viteri and Linares, 2017; Viteri and Linares, 2019; Viteri et al., 2019) may increase the levels of ASB resistance. Likewise, multiple crosses with the UPR-Mp BL would be recommended to incorporate more desirable resistant genes/QTL for the development of new germplasms in the near future. Singh et al. (2017) incorporated higher levels of resistance (scores <3) to white mold in PRA155 by the use of six parents from Andean and Middle American beans (50% of genetic contribution) crossed with A 195 (50% of the remaining contribution).

Since the levels of pathogenicity between *M. phaseolina* isolates can vary between countries/regions (Marquez et al., 2021) and even in the same country [e.g., Mexico (Mayek et al., 2001a) and Puerto Rico (Miklas et al., 1998a; Viteri and Linares, 2017)], future studies should be focused on the screening of the UPR-Mp BL against multiple isolates. This would help to identify if these BL have any specific or broad spectrum of durable partial resistance. For example, Singh et al. (2014) and Viteri et al. (2015) identified that the common bean BL SE152-6, SE153-7, and SE155-9 were resistant during the entire reproductive stage to ARS12D, CO463, ND710, and NY133 *S. sclerotiorum* isolates from Argentina and the United States using the same methodology reported in this study. Furthermore, the expression profiling of resistant genes/QTL at different times after the inoculation of *M. phaseolina* isolates should be an important topic to explore in future research.

In summary, the UPR-Mp BL had an intermediate response to ASB in the field and the greenhouse. These BL may be used to improve black, cranberry, and white common bean germplasms (which are in the 10 market classes that dominate the common bean production in the United States; Miklas and Riley, 2012) and/or other market classes.

## Data availability statement

The raw data supporting the conclusions of this article will be made available by the authors, without undue reservation.

## Author contributions

**DV:** Supervision, conceptualization, data collecting, methodology, investigation, project administration, review and editing, validation, writing-original draft. **AL:** Data collecting, methodology, supervision, resources, review and editing. All authors contributed to the article and approved the submitted version.

## Acknowledgments

We thank the USDA-NIFA Regional Project S-009 (Award number: 1017544) for supporting the funds of this research. The authors also thank Luis Cabán, Zoralys Miranda, Arturo Luciano, and Roberto Vázquez for their support in the field and greenhouse activities.

## Conflict of interest

The authors declare that the research was conducted in the absence of any commercial or financial relationships that could be construed as a potential conflict of interest.

## Publisher’s note

All claims expressed in this article are solely those of the authors and do not necessarily represent those of their affiliated organizations, or those of the publisher, the editors and the reviewers. Any product that may be evaluated in this article, or claim that may be made by its manufacturer, is not guaranteed or endorsed by the publisher.

## Abbreviations

ASB, ashy stem blight; BL, breeding line/lines; QTL, quantitative trait loci/locus.

## References

[B1] Ambachew D. Joshua J. Mmbaga M. T. Blair M. W. (2021). Sources of resistance to common bacterial blight and charcoal rot disease for the production of mesoamerican common beans in the southern united states. Plants 10, 998. doi: 10.3390/plants10050998 34067661 PMC8156677

[B2] Beaver J. S. Estévez de Jensen C. Lorenzo-Vásquez G. González A. Martínez H. Porch T. G. (2018). Registration of ‘Bella’ white-seeded common bean cultivar. J. Plant Regist. 12, 190–193. doi: 10.3198/jpr2017.05.0029crc

[B3] Beaver J. S. Estévez de Jensen C. Miklas P. N. Porch T. G. (2020). Contributions in Puerto Rico to bean, *Phaseolus* spp., research. J. Agric. Univ. Puerto Rico 104, 43–111. doi: 10.46429/jaupr.v104i1.18287

[B4] Beebe S. E. Rao I. M. Blair M. W. Acosta-Gallegos J. A. (2013). Phenotyping common beans for adaptation to drought. Front. Physiol. 4. doi: 10.3389/fphys.2013.00035 PMC3589705 23507928

[B5] Cordeiro G. M. (1983). Improved likelihood ratio statistics for generalized linear models. J. R. Stat. Soc. 45, 404–413. doi: 10.1111/j.2517-6161.1983.tb01269.x

[B6] Echavez-Badel R. Beaver J. S. (1987). Resistance and susceptibility of beans to ashy stem blight. J. Agric. Univ. Puerto Rico 72, 403–406. doi: 10.46429/jaupr.v71i4.6988

[B7] Frahm M. A. Rosas J. C. Mayek N. López-Salinas E. Acosta-Gallegos J. A. Kelly J. D. (2004). Breeding beans for resistance to terminal drought in the lowland tropics. Euphytica 136, 223–232. doi: 10.1023/B:euph.0000030671.03694.bb

[B8] Islam S. Haque S. Islam M. M. Emdad E. M. Halim A. Hossen Q. M.. (2012). Tools to kill: genome of one of the most destructive plant pathogenic fungi *Macrophomina phaseolina* . BMC Genomics 13, 1–16. doi: 10.1186/1471-2164-13-493 22992219 PMC3477038

[B9] Kaur S. Singh G. Kaur S. Vallad G. Chand R. Chauhan V. (2012). Emerging phytopathogen *Macrophomina phaseolina*: biology, economic importance and current diagnostic trends. Crit. Rev. Microbiol. 38, 136151. doi: 10.3109/1040841X.2011.640977 22257260

[B10] Kelly J. D. Varner G. B. O’Boyle P. Long B. (2009). Registration of ‘Zorro’ black bean. J. Plant Regist. 3, 226–230. doi: 10.3198/jpr2008.12.0730crc

[B11] Marquez N. Guiachero M. L. Declerk S. Ducasse D. A. (2021). *Macrophomina phaseolina*: general characteristics of pathogenicity and methods of control. Front. Plant Sci. 22. doi: 10.3389/fpls.2021.634397 PMC8100579 33968098

[B12] Mayek N. García R. López C. Acosta J. A. Simpson J. (2002). Water relations, histopathology and growth of common bean (*Phaseolus vulgaris* L.) during pathogenesis of *Macrophomina phaseolina* under drought stress. Physiol. Mol. Plant Pathol. 60, 185–195. doi: 10.1006/pmpp.2001.0388

[B13] Mayek N. López E. Cumpián J. Acosta J. A. (2009). Herencia de la resistencia de campo a *Macrophomina phaseolina* (Tassi) Goid. en líneas endogámicas recombinantes de frijol común (*Phaseolus vulgaris* L.). Rev. Mexicana Fitopatología 27, 1–10.

[B14] Mayek N. López C. González M. García R. Acosta J. Martínez de la Vega O.. (2001a). Variability of Mexican isolates of *Macrophomina phaseolina* based on pathogenesis and AFLP genotype. Physiol. Mol. Plant Pathol. 59, 257–264. doi: 10.1006/pmpp.2001.0361

[B15] Mayek N. López C. López E. Cumpián J. Acosta J. A. (2001b). Resistance to *Macrophomina phaseolina* (Tassi) goid. in common bean under field conditions in méxico. Agrociencia 35, 649–661.

[B16] Mayek N. López C. López E. Cumpián J. Torres I. C. Padilla J. S.. (2003). Effect of *Macrophomina phaseolina* (Tassi) Goid. on grain yield of common beans (*Phaseolus vulgaris* L.) and its relationship with yield stability parameters. Rev. Mexicana Fitopatología 21, 168175.

[B17] McIntosh M. S. (1983). Analysis of combined experiments. Agron. J. 75, 153–155. doi: 10.2134/agronj1983.00021962007500010041x

[B18] Méndez R. Reyes M. H. Hernández S. López E. Cumpián J. Cantú M. A.. (2017). Identification and mapping of QTLs associated with resistance to *Macrophomina phaseolina* and drought stress in common beans. Annu. Rep. Bean Improv. Coop. 60, 23–24.

[B19] Miklas P. N. Delorme R. (2003). Identification of QTL conditioning resistance to white mold in snap bean. J. Am. Soc. Hortic. Sci. 128, 564–570. doi: 10.21273/JASHS.128.4.0564

[B20] Miklas P. N. Riley R. (2012). Registration of ‘Crimson’ cranberry bean. J. Plant Regist. 6, 11–14. doi: 10.3198/jpr2011.05.0251crc

[B21] Miklas P. N. Schwartz H. F. Salgado M. O. Nina R. Beaver J. (1998a). Reaction of select tepary bean to ashy stem blight and *Fusarium* wilt. HortScience 33, 136–139.

[B22] Miklas P. N. Stone V. Urrea C. Johnson E. Beaver J. (1998b). Inheritance and QTL analysis of field resistance to ashy stem blight in common bean. Crop Sci. 38, 916–921. doi: 10.2135/cropsci1998.0011183X003800040004x

[B23] Muñoz M. A. Lugo W. I. Santiago C. Matos M. Rios S. Lugo J. (2018). “Taxonomic classification of the soils of Puerto Rico 2017,” in Bulletin 313 (San Juan: Agricultural Experimental Station), 14–26.

[B24] Olaya G. Abawi G. S. Weeden N. F. (1996). Inheritance of the resistance to *Macrophomina phaseolina* and identification of RAPD markers linked to the resistance genes in beans. Phytopathology 86, 674–679. doi: 10.1094/Phyto-86-674

[B25] Pastor-Corrales M. A. Abawi G. S. (1988). Reactions of selected bean accessions to infection by *M. phaseolina* . Plant Dis. 72, 39–41. doi: 10.1094/PD-72-0039

[B26] Porch T. G. Urrea C. A. Beaver J. S. Valentin S. Peña P. A. Smith J. R. (2012). Registration of TARS-MST1 and SB-DT1 multiple-stress-tolerant black bean germplasm. J. Plant Regist. 6, 75–80. doi: 10.3198/jpr2010.08.0501crg

[B27] Saladin F. Arnaud-Santana E. Nin J. C. Godoy-Lutz G. Beaver J. S. Coyne D. P. (2000). Registration of ‘PC 50’ red mottled bean. Crop Sci. 40, 858.

[B28] SAS Institute (2012). SAS/STAT user’s manual, version 9.4 (Cary, NC: SAS Institute).

[B29] Schwartz H. F. Singh S. P. (2013). Breeding common bean for resistance to white mold: a review. Crop Sci. 53, 1832–1844. doi: 10.2135/cropsci2013.02.0081

[B30] Singh S. P. (1982). A key for identification of different growth habits of *Phaseolus vulgaris* L. Annu. Rep. Bean Improv. Coop. 25, 92–95.

[B31] Singh S. P. (1994). Gamete selection for simultaneous improvement of multiple traits in common bean. Crop Sci. 34, 352–355. doi: 10.2135/cropsci1994.011183X003400020008x

[B32] Singh S. P. Schwartz H. F. (2010). Breeding common bean for resistance to diseases: a review. Crop Sci. 50, 2199–2223. doi: 10.2135/cropsci2009.03.0163

[B33] Singh S. P. Schwartz H. F. Terán H. Centeno C. Otto C. (2016). Registration of common bean pinto PRP 153 and VCP 13 with high levels of broad-spectrum white mold resistance. J. Plant Regist. 10, 291–295. doi: 10.3198/jpr2016.01.0003crg

[B34] Singh S. P. Schwartz H. F. Terán H. Centeno C. Otto C. (2017). Large-Seeded common bean PRA 152, PRA 154, and PRA 155 with high levels of broad-Spectrum white mold resistance. J. Plant Regist. 11, 305–310. doi: 10.3198/jpr2016.01.0003crg

[B35] Singh S. P. Schwartz H. F. Terán H. Viteri D. Otto C. (2014). Pyramiding white mould resistance between and within common bean gene pools. Can. J. Plant Sci. 94, 947–954. doi: 10.4141/cjps2013-321

[B36] Singh S. P. Terán H. Gutiérrez A. (2001). Registration of SEA 5 and SEA 13 drought tolerant dry bean germplasm. Crop Sci. 41, 276–277. doi: 10.2135/cropsci2001.411276x

[B37] Singh S. P. Terán H. Lema M. Schwartz H. F. Miklas P. N. (2007). Registration of white mold resistant dry bean germplasm line A 195. J. Plant Regist. 1, 62–63. doi: 10.3198/jpr2006.10.0643crg

[B38] Urrea C. A. Yonts C. D. Lyon D. J. Koehler A. E. (2009). Selection for drought tolerance in dry bean derived from the mesoamerican gene pool in western Nebraska. Crop Sci. 49, 2005–2010. doi: 10.2135/cropsci2008.12.0694

[B39] Viteri D. M. Linares A. M. (2017). Reaction of *Phaseolus* spp. genotypes to ashy stem blight caused by *Macrophomina phaseolina* . Euphytica 213, 199. doi: 10.1007/s10681-017-1989-y

[B40] Viteri D. M. Linares A. M. (2019). Inheritance of ashy stem blight resistance in Andean common bean cultivars ‘Badillo’ and ‘PC 50’ and genetic relationship between Andean A 195 and ‘PC 50’. Euphytica 215, 12. doi: 10.1007/s10681-019-2336-2

[B41] Viteri D. M. Linares A. M. (2022). Agronomic performance of common and tepary bean genotypes and their response to ashy stem blight in isabela and lajas, Puerto Rico. Legume Sci. 4, e118. doi: 10.1002/leg3.118

[B42] Viteri D. M. Linares A. M. Urrea C. A. (2019). Effect of multiple inoculations of an aggressive *Macrophomina phaseolina* isolate for screening common bean genotypes under high temperatures. Annu. Rep. Bean Improv. Coop. 62, 17–18.

[B43] Viteri D. M. Otto K. Terán H. Schwartz H. F. Singh S. P. (2015). Use of four *Sclerotinia sclerotiorum* isolates of different aggressiveness, three inoculations per plant, and delayed multiple evaluations to select common beans with high levels of white mold resistance. Euphytica 204, 457–472. doi: 10.1007/s10681-015-1366-7

